# Occupational epidemiological characteristics of noise-induced hearing loss and the impact of combined exposure to noise and dust on workers’ hearing—a retrospective study

**DOI:** 10.3389/fpubh.2024.1488065

**Published:** 2024-10-30

**Authors:** Bin Zhou, Jiaxiang Zhang

**Affiliations:** ^1^The Third People’s Hospital of Hefei, Hefei Third Clinical College of Anhui Medical University, Hefei, Anhui, China; ^2^School of Public Health, Anhui Medical University, Hefei, Anhui, China

**Keywords:** noise, NIHL, epidemiological characteristics, dust, combined exposure

## Abstract

**Introduction:**

The aim of the study was to investigate the occupational epidemiological characteristics of hearing loss among noise-exposed workers through a cross-sectional study and to explore the impact of combined noise and dust exposure on workers’ hearing loss through a longitudinal study.

**Results:**

This cross-sectional study revealed that the risk of speech-frequency hearing loss increases with age (OR = 1.096, 95%CI = 1.081–1.111). Independent factors influencing high-frequency hearing loss include sex, age, hazardous factors, industry category, and enterprise size. Scientific research and technical services (OR = 1.607, 95%CI = 1.111–2.324), wholesale and retail (OR = 2.144, 95%CI = 1.479–3.107), manufacturing (OR = 1.907, 95%CI = 1.429–2.545), and other industries (OR = 1.583, 95%CI = 1.002–2.502) are risk factors for high-frequency hearing loss, whereas being female (OR = 0.297, 95%CI = 0.236–0.373) is a protective factor against high-frequency hearing loss. Independent factors influencing occupational noise-induced hearing loss include sex, working age, hazardous factors, industry category, smoking, and drinking, with the risk of occupational noise-induced hearing loss increasing with working age (OR = 1.045, 95%CI = 1.031–1.058). Noise and dust work (OR = 1.271, 95%CI = 1.011–1.597), other work (OR = 0.619, 95%CI = 0.479–0.800), manufacturing (OR = 2.085, 95%CI = 1.336–3.254), other industries (OR = 2.063, 95%CI = 1.060–4.012), occasional smokers (OR = 0.863, 95%CI = 0.652–1.142), regular smokers (OR = 1.216, 95% CI = 0.987–1.497), and excessive drinkers (OR = 2.171, 95%CI = 1.476–3.193) are risk factors for occupational noise-induced hearing loss, whereas being female (OR = 0.496, 95%CI = 0.347–0.709) is a protective factor against occupational noise-induced hearing loss. The longitudinal study revealed differences in pure-tone hearing threshold test results at 500 Hz, 1,000 Hz, 3,000 Hz, 4,000 Hz, and 6,000 Hz in both ears before and after enrollment among noise-exposed workers (*p* < 0.05). Combined noise and dust exposure (OR = 4.660, 95%CI = 1.584–13.711), 1st year (OR = 1.540, 95%CI = 1.128–2.103), 2nd year (OR = 1.994, 95%CI = 1.409–2.821), and 3rd year (OR = 1.628, 95%CI = 1.170–2.264) were risk factors for high-frequency hearing loss.

**Discussion:**

Combined noise and dust exposure is a risk factor for occupational noise-induced hearing loss. Additionally, occupational noise-induced hearing loss is influenced by gender, working age, enterprise industry category, smoking, and drinking. Employers should enhance occupational health management and improve workers’ occupational health literacy, with a particular focus on older male workers of long working age, and those with unhealthy habits. Combined exposure to noise and dust may have a synergistic effect on causing high-frequency hearing loss, and comprehensive protective measures should be implemented for workers exposed to both.

## 1 Introduction

The World Hearing Report published by the World Health Organization (WHO) shows that currently, over 1.5 billion people worldwide suffer from hearing impairment. Occupational noise exposure is the primary cause of hearing impairment in adults (1). Noise-induced hearing loss (NIHL) has become a global public health issue (2, 3). As a major manufacturing country, China has numerous industrial enterprises with severe noise hazards; according to a national occupational disease hazard survey conducted by the National Health Commission in 2020, 88.81% of enterprises were found to have noise hazards. Among the 8.7 million workers exposed to occupational disease hazards, 71.95% were exposed to noise (4). Occupational noise exposure is one of the most serious occupational diseases in China (5), with research data showing that the rate of excessive personal noise exposure is 42.9% in the manufacturing of railways, ships, aerospace, and other transport equipment and 36.4% in the automotive manufacturing industry (6).

As a central region province, Anhui has taken over industrial transfer from China’s eastern coastal areas. In recent years, Hefei, the core city, has been committed to industrial upgrading and economic transformation, with leading industries shifting from traditional sectors to emerging industries such as new materials, new energy, and high-end equipment manufacturing. The severe noise hazards caused by industrial transformation require significant attention.

Long-term exposure to occupational noise exceeding an 8-h equivalent sound level of 85 dB(A), resulting in sensorineural hearing loss, is known as occupational noise-induced deafness (ONID). National health statistics show that the number of new cases has increased in recent years. Occupational noise exposure can lead to auditory system damage, such as temporary threshold shift (TTS), permanent threshold shift (PTS), and tinnitus, as well as nonauditory system damage, including emotional and cognitive disorders, sleep disturbances, and cardiovascular diseases (7). The pathological changes in the cochlea caused by noise exposure mainly include damage to and death of hair cells, loss of ribbon synapses in the cochlea, degeneration of spiral ganglion neuron fibers, and damage to the cochlear stria vascularis (8–11). TTS is closely related to the inner ear, and it is associated with reduced cochlear blood flow, cochlear dysfunction, and changes in inner ear potentials caused by stereocilia disarray. The most common cause of PTS is the damage and death of cochlear hair cells. In China, ONID has become the second most common occupational disease (12, 13). NIHL is a complex disease induced by both environmental and genetic factors (14, 15), with noise characteristics, noise intensity, noise peaks, exposure duration, and individual susceptibility influencing its development (3, 16, 17). NIHL is considered irreversible, and there are currently no FDA-approved drugs for its treatment (18, 19). Steroids are often used clinically, but their efficacy is not significant. Therefore, understanding the distribution characteristics of NIHL is crucial for monitoring and controlling noise hazards and preventing and treating occupational noise-induced hearing loss.

Some studies have shown that in addition to noise, combined exposure to occupational chemicals and some physical factors can also increase the degree of damage to the auditory system (20). The development of productivity and the promotion of new materials, technologies, and processes have made workplace hazard exposure increasingly complex, with combined exposure to multiple hazards becoming the norm. Research has shown that combined exposure to noise and organic solvents such as benzene, xylene, and styrene can increase the risk of hearing loss among workers (21–24). Noise and dust are the most severe occupational disease hazards for workers, making it important to explore the effects of combined exposure to noise and dust on the auditory system in the prevention and treatment of occupational noise-induced hearing loss. This large-scale cross-sectional study aimed to understand the distribution characteristics of hearing loss among noise-exposed workers in Hefei. We also explored the impact of combined exposure to noise and dust on hearing loss through longitudinal research.

## 2 Object and methods

### 2.1 Object

This study includes both cross-sectional and longitudinal studies that target personnel undergoing occupational health examinations for noise exposure at the Hefei Occupational Disease Prevention and Control Hospital. All participants were scheduled for examinations according to GBZ188-2014 “Technical Specifications for Occupational Health Surveillance.” A cross-sectional study was conducted to investigate the distribution characteristics of hearing loss among 7,470 noise-exposed workers from July 2020 to June 2021. A longitudinal study was conducted by selecting three manufacturing enterprises, retrieving their occupational health assessment reports, and dividing 362 workers into a noise exposure group and a noise and dust coexposure group on the basis of their exposure to hazardous factors. The pure-tone audiometry results of the two groups from 2017 to 2020 were collected to analyze the impact of combined exposure to noise and dust on workers’ hearing loss.

The exclusion criteria included a history of middle ear diseases; familial or traumatic deafness; recent use of ototoxic drugs such as streptomycin or gentamicin; suspected simulated hearing loss; and refusal to participate in the study.

### 2.2 Methods

#### 2.2.1 Questionnaire survey

A questionnaire was designed for face-to-face surveys with the subjects. The main contents of the questionnaire included the following: (1) basic information, including name, sex, age, and contact information; (2) occupational history, including workshop, type of work, duration of hazardous exposure, exposure to occupational disease hazards, and use of personal protective equipment; (3) personal life history, including smoking history and drinking history; and (4) marital and reproductive history, past medical history, family genetic history, and medication history.

#### 2.2.2 Variable grouping

The subjects were grouped according to variables such as sex, age, duration of hazardous exposure, hazardous factors, industry category, enterprise size, smoking, and drinking. Grouping was based on exposure to hazardous factors: workers exposed solely to noise were categorized into the noise exposure group, and those exposed to both noise and dust (all types of industrial dust) were categorized into the noise-dust group. Those engaged in welding work and exposed to noise, welding fumes, ultraviolet radiation, manganese and its compounds, and nitrogen-containing compounds were categorized into the welding group. Those exposed to both noise and high temperatures were categorized into the noise-high temperature group. The remaining workers exposed to noise and other hazardous factors were categorized into the other group. Industry categories were grouped according to GB/T 4754–2017 “Industrial Classification for National Economic Activities.” Enterprise sizes were grouped according to the “Statistical Classification of Large, Medium, Small, and Microenterprises” issued by the National Bureau of Statistics of China (25). The subjects were divided into nonsmokers, occasional smokers (smoking more than 4 times per week but averaging less than one cigarette per day), and regular smokers (smoking more than one cigarette per day for more than 6 months). In accordance with the “Dietary Guidelines for Chinese Residents (26),” the subjects were divided into nondrinkers, moderate drinkers (averaging no more than 15 g of alcohol per day), and excessive drinkers (averaging more than 15 g of alcohol per day) (26).

#### 2.2.3 Pure tone audiometry

Pure tone audiometry was conducted according to GB/T16403-1996 “Acoustics: Methods for Audiometry, Basic Pure Tone Air and Bone Conduction Audiometry,” using a Danish Interacoustics AD229b instrument operated by professionally trained medical personnel. The pure tone air conduction hearing thresholds of the study subjects were measured at speech frequencies (500 Hz, 1,000 Hz, 2000 Hz) and high frequencies (3,000 Hz, 4,000 Hz, 6,000 Hz). The subjects were required to avoid noisy environments for at least 48 h before the test.

#### 2.2.4 Definition of hearing loss results

According to the WHO’s classification standards for hearing loss, a threshold of ≤25 dB at each tested frequency is considered normal. The test results were adjusted for sex and age on the basis of the statistical distribution in GB/T 7582: “Statistical distribution of hearing threshold and age.” The definition of noise-induced hearing loss was based on GBZ49-2014 “Diagnosis of occupational noise-induced deafness.”

1. Definition of speech-frequency hearing loss (SFHL): the average thresholds at 500 Hz, 1,000 Hz, and 2000 Hz in both ears were calculated, with results >25 dB HL defined as SFHL.
2. Definition of High-frequency hearing loss (HFHL): the average thresholds at 3000 Hz, 4,000 Hz, and 6,000 Hz in both ears were calculated, with results >25 dB HL defined as HFHL.
3. Definition of Occupational noise-induced hearing loss (ONIHL): the average thresholds at 3000 Hz, 4,000 Hz, and 6,000 Hz in both ears were calculated, with results ≥40 dB HL defined as ONIHL.

#### 2.2.5 Quality control

Unified training was provided to the investigators before the survey began. Professional medical personnel conducted pure tone audiometry on the subjects according to standardized procedures. Instruments and equipment were calibrated and verified according to relevant regulations and standards. The research data were entered by two people to check for consistency.

### 2.3 Statistical analysis

A database was established via Excel software, and the statistical analyses were conducted via SPSS 29.0 software. Categorical data are expressed as rates and composition ratios, and differences were analyzed via the chi-square test. Logistic regression was used to analyze the influencing factors of various types of hearing loss (inclusion criterion *p* < 0.10), with model fit assessed by the Hosmer–Lemeshow test. Continuous data were tested for normality; normally distributed data are expressed as the mean ± standard deviation (X ± S), whereas nonnormally distributed data are expressed as the median (M) and interquartile range (QR). Repeated measures data were analyzed via repeated-measures ANOVA or the Friedman test. The influencing factors of various types of hearing loss were analyzed via generalized estimating equations. The significance level for tests was set at *p* < 0.05.

## 3 Results

### 3.1 Baseline data of the study subjects

A total of 7,470 individuals aged 17–70 years, with an average age of 35.70 ± 9.89 years, were included in this cross-sectional study. There were 6,338 males (84.85%) and 1,132 females (15.15%). The noise exposure group included 2,232 individuals (29.88%), the noise and dust group included 2066 individuals (27.66%), the welding group included 749 individuals (10.03%), the noise and high-temperature group included 200 individuals (2.68%), and the other groups included 2,223 individuals (29.76%). The manufacturing group included 5,775 individuals (77.31%), the scientific research and technical services group 505 individuals (6.76%), the energy production and supply group 504 individuals (6.75%), the wholesale and retail group 467 individuals (6.25%), and the other industries group 219 individuals (2.93%). Large enterprises included 2,948 individuals (39.46%), medium-sized enterprises 1773 individuals (23.74%), small enterprises 2,346 individuals (31.41%), microenterprises 296 individuals (3.96%), and enterprises of unknown size 107 individuals (1.43%).

The longitudinal study on hearing loss in noise-exposed workers included a total of 362 individuals, all of whom were male. The ages of the participants ranged from 25 to 56 years, with an average age of 35.75 ± 5.48 years. There were 86 individuals (23.76%) in the noise exposure group and 276 individuals (76.24%) in the noise and dust groups. All 362 individuals wore personal protective equipment while working; those exposed to noise wore 3 M noise-reducing earplugs and earmuffs, whereas those exposed to dust wore 3 M dust masks.

### 3.2 Current status and influencing factors of hearing loss among noise-exposed workers

The distribution of SFHL significantly differed across variables such as age, hazard factors, enterprise size, and alcohol consumption. The prevalence of SFHL was 0.81% in the <30 years group, 1.88% in the 30–39 years group, 5.69% in the 40–49 years group, and 12.19% in the ≥50 years group. The prevalence of SFHL in the noise exposure group, noise and dust group, welding group, noise and high-temperature group, and other groups was 3.23, 4.84, 2.54, 4.50, and 3.01%, respectively. The prevalence rates of SFHL in large enterprises, medium–sized enterprises, small enterprises, microenterprises, and enterprises of unknown size were 1.80, 3.44, 5.71, 5.41, and 2.80%, respectively. The prevalence of SFHL among those who never drank, drank moderately, and drank excessively was 3.30, 3.42, and 10.43%, respectively (Table 1). Logistic regression analysis revealed that age was an independent influencing factor for SFHL. For noise-exposed workers, the risk of developing SFHL increased by a factor of 1.096 (1.081–1.111) for each additional year of age (Table 2).

**Table 1:** Distribution of different types of hearing loss across various variables.

Variables	Total number of workers	SFHL			HFHL			ONIHL		
		Number (*n*%)	*χ* ^2^	*p*	Number (*n*%)	*χ* ^2^	*p*	Number (*n*%)	*χ* ^2^	*p*
Sex			0.622	0.430		63.027	0.000		25.588	0.000
Male	6,338	222 (3.50%)			1,373 (21.66%)			497 (7.84)		
Female	1,132	45 (3.98%)			129 (11.40%)			41 (3.62)		
Age (years)			290.013	0.000		742.496	0.000		532.313	0.000
<30	2,107	17 (0.81%)			175 (8.31%)			34 (1.61)		
30~	3,031	57 (1.88%)			440 (14.52%)			110 (3.63)		
40~	1,405	80 (5.69%)			479 (34.09%)			195 (13.88%)		
50~	927	113 (12.19%)			408 (44.01%)			199 (21.47%)		
Years of exposure to hazards (years)			7.443	0.114		68.645	0.000		45.312	0.000
<3	3,049	104 (3.41%)			541 (17.74%)			195 (6.40%)		
3~	1,647	60 (3.64%)			326 (19.79%)			107 (6.50%)		
7~	1,217	47 (3.86%)			248 (20.38%)			90 (7.40%)		
11~	981	26 (2.65%)			198 (20.18%)			65 (6.63%)		
15~	576	30 (5.21%)			189 (32.81%)			81 (14.06%)		
Hazard factors			15.256	0.004		76.697	0.000		31.812	0.000
Noise	2,232	72 (3.23%)			390 (17.47%)			151 (6.77%)		
Noise and dust	2066	100 (4.84%)			527 (25.51%)			189 (9.15%)		
Welding	749	19 (2.54%)			184 (24.57%)			70 (9.35%)		
Noise and high temperature	200	9 (4.50%)			39 (19.50%)			14 (7.00%)		
Other groups	2,223	67 (3.01%)			362 (16.28%)			114 (5.13%)		
Industry category			0.075	0.999		14.033	0.007		9.288	0.054
Energy production and supply	504	18 (3.57%)			72 (14.29%)			23 (4.56%)		
Scientific research and technical services	505	17 (3.37%)			96 (19.01%)			32 (6.34%)		
Wholesale and retail	467	17 (3.64%)			98 (20.99%)			26 (5.57%)		
Manufacturing	5,775	207 (3.58%)			1,183 (20.48%)			440 (7.62%)		
Other industries	219	8 (3.65%)			53 (24.20%)			17 (7.76%)		
Enterprise size			61.251	0.000		128.605	0.000		117.742	0.000
Large	2,948	53 (1.80%)			453 (15.37%)			118 (4.00%)		
Medium	1773	61 (3.44%)			312 (17.60%)			112 (6.32%)		
Small	2,346	134 (5.71%)			629 (26.81%)			260 (11.08%)		
Micro	296	16 (5.41%)			86 (29.05%)			40 (13.51%)		
Unknown	107	3 (2.80%)			22 (20.56%)			8 (7.48%)		
Smoking			4.986	0.083		36.495	0.000		24.971	0.000
Non-smokers	4,120	148 (3.59%)			740 (17.96%)			255 (6.19%)		
Occasional smokers	1,166	30 (2.57%)			230 (19.73%)			75 (6.43%)		
Regular smokers	2,184	89 (4.08%)			532 (24.36%)			208 (9.52%)		
Drinking			32.487	0.000		83.811	0.000		48.771	0.000
Non-drinkers	3,880	128 (3.30%)			670 (17.27%)			234 (6.03%)		
Moderate drinkers	3,360	115 (3.42%)			740 (22.02%)			263 (7.83%)		
Excessive drinkers	230	24 (10.43%)			92 (40.00%)			41 (17.83%)		

**Table 2:** Logistic regression analysis of factors affecting SFHL.

Variables	*β*	S.E.	Wald	*p*	OR	95%CI
Age (years)	0.092	0.007	175.486	0.000	1.096	1.081–1.111
Hazard factors			0.624	0.960		
Noise	–	–	–	–	1.000	–
Noise and dust	−0.018	0.167	0.012	0.913	0.982	0.708–1.362
Welding	−0.072	0.270	0.070	0.791	0.931	0.548–1.581
Noise and high temperature	−0.212	0.370	0.327	0.567	0.809	0.392–1.671
Other groups	0.050	0.178	0.080	0.777	1.052	0.742–1.491
Enterprise size			7.924	0.094		
Large	–	–	–	–	1.000	–
Medium	0.457	0.196	5.455	0.020	1.579	1.076–2.317
Small	0.471	0.177	7.038	0.008	1.601	1.131–2.267
Micro	0.321	0.308	1.086	0.297	1.379	0.754–2.523
Unknown	0.292	0.615	0.226	0.634	1.340	0.401–4.471
Smoking			0.200	0.905		
Non-smokers	–	–	–	–	1.000	–
Occasional smokers	−0.095	0.218	0.189	0.664	0.910	0.593–1.394
Regular smokers	−0.006	0.155	0.001	0.970	0.994	0.733–1.348
Drinking			4.507	0.105		
Non-drinkers	–	–	–	–	1.000	–
Moderate drinkers	0.188	0.146	1.655	0.198	1.207	0.906–1.607
Excessive drinkers	0.528	0.261	4.086	0.043	1.695	1.016–2.827

The distribution of HFHL showed significant differences across variables such as sex, age, years of service, hazard factors, industry category, enterprise size, smoking, and alcohol consumption. The prevalence rates of HFHL in males and females were 21.66 and 11.40%, respectively. The prevalence of HFHL was 8.31% in the <30 years group, 14.52% in the 30–39 years group, 34.09% in the 40–49 years group, and 44.01% in the ≥50 years group. The prevalence of HFHL was 17.74% in the <3 years group, 19.79% in the 3–6 years group, 20.38% in the 7–10 years group, 20.18% in the 11–14 years group, and 32.81% in the ≥15 years group. The prevalence of HFHL in the noise exposure group, noise and dust group, welding group, noise and high-temperature group, and other groups was 17.47, 25.51, 24.57, 19.50, and 16.28%, respectively. The prevalence rates of HFHL in the energy production and supply industry, scientific research and technical services industry, wholesale and retail industry, manufacturing industry, and other industries were 14.29, 19.01, 20.99, 20.48, and 24.20%, respectively. The prevalence of HFHL in large enterprises, medium–sized enterprises, small enterprises, microenterprises, and enterprises of unknown size was 15.37, 17.60, 26.81, 29.05, and 20.56%, respectively. The prevalence of HFHL among those who never smoked, occasionally smoked, and regularly smoked was 17.96, 19.73, and 24.36%, respectively. The prevalence of HFHL among those who never drank, drank moderately, and drank excessively was 17.27, 22.02, and 40.00%, respectively (Table 1). The logistic regression analysis results revealed that sex, age, hazard factors, industry category, and enterprise size were independent influencing factors for HFHL. The risk of developing HFHL in females was 0.297 (0.236–0.373) times greater than that in males. For noise-exposed workers, the risk of developing HFHL increased by a factor of 1.088 (1.080–1.096) for each additional year of age. The risk of developing HFHL in the welding group was 1.440 (1.150–1.802) times greater than that in the noise exposure group. The risk of developing HFHL in the scientific research and technical services industry, wholesale and retail industry, manufacturing industry, and other industries was 1.607 (1.111–2.324), 2.144 (1.479–3.107), 1.907 (1.429–2.545), and 1.583 (1.002–2.502) times greater than that in the energy production and supply industry, respectively. The risk of developing HFHL for workers in small enterprises was 1.434 (1.219–1.687) times greater than that for workers in large enterprises (Table 3).

**Table 3:** Logistic regression analysis of factors affecting HFHL.

Variables	*β*	S.E.	Wald	*p*	OR	95% CI
Sex			–	–		
Male	–	–	–	–	1.000	–
Female	−1.215	0.117	108.374	0.000	0.297	0.236–0.373
Age (years)	0.08	0.004	530.599	0.000	1.088	1.080–1.096
Years of exposure to hazards (years)	−0.002	0.005	0.180	0.671	0.998	0.988–1.008
Hazard factors			25.231	0.000		
Noise	–	–	–	–	1.000	–
Noise and dust	0.052	0.085	0.376	0.540	1.053	0.892–1.243
Welding	0.364	0.114	10.143	0.001	1.440	1.150–1.802
Noise and high temperature	−0.383	0.207	3.422	0.064	0.682	0.454–1.023
Other groups	−0.148	0.087	2.888	0.089	0.862	0.727–1.023
Industry category			22.708	0.000		
Energy production and supply	–	–	–	–	1.000	–
Scientific research and technical services	0.474	0.188	6.359	0.012	1.607	1.111–2.324
Wholesale and retail	0.763	0.189	16.230	0.000	2.144	1.479–3.107
Manufacturing	0.646	0.147	19.213	0.000	1.907	1.429–2.545
Other industries	0.460	0.233	3.877	0.049	1.583	1.002–2.502
Enterprise size			20.674	0.000		
Large	–	–	–	–	1.000	–
Medium	0.100	0.088	1.288	0.256	1.105	0.930–1.313
Small	0.361	0.083	18.959	0.000	1.434	1.219–1.687
Micro	0.278	0.160	3.013	0.083	1.320	0.965–1.806
Unknown	0.363	0.277	1.723	0.189	1.438	0.836–2.473
Smoking			1.157	0.561		
Non-smokers	–	–	–	-	1.000	–
Occasional smokers	0.084	0.095	0.781	0.377	1.088	0.902–1.312
Regular smokers	0.068	0.075	0.806	0.369	1.070	0.923–1.240
Drinking			1.824	0.402		
Non-drinkers	–	–	–	–	1.000	–
Moderate drinkers	0.094	0.071	1.776	0.183	1.099	0.957–1.262
Excessive drinkers	0.091	0.161	0.322	0.570	1.096	0.799–1.502

The distribution of ONIHL scores significantly differed across variables such as sex, age, years of service, hazard factors, enterprise size, smoking, and alcohol consumption. The prevalence rates of ONIHL in males and females were 7.84 and 3.62%, respectively. The prevalence of ONIHL was 1.61% in the <30 years group, 3.63% in the 30–39 years group, 13.88% in the 40–49 years group, and 21.47% in the ≥50 years group. The prevalence of ONIHL was 6.40% in the <3 years group, 6.50% in the 3–6 years group, 7.40% in the 7–10 years group, 6.63% in the 11–14 years group, and 14.06% in the ≥15 years group. The prevalence rates of ONIHL in the noise exposure group, noise and dust group, welding group, noise and high-temperature group, and other groups were 6.77, 9.15, 9.35, 7.00, and 5.13%, respectively. The prevalence rates of ONIHL in large enterprises, medium–sized enterprises, small enterprises, microenterprises, and enterprises of unknown size were 4.00, 6.32, 11.08, 13.51, and 7.48%, respectively. The prevalence of ONIHL among those who never smoked, occasionally smoked, and regularly smoked was 6.19, 6.43, and 9.52%, respectively. The prevalence of ONIHL among those who never drank, drank moderately, and drank excessively was 6.03, 7.83, and 17.83%, respectively (Table 1). The logistic regression equation related to ONIHL showed poor model fit according to the Hosmer–Lemeshow test (*p* < 0.01). The included factors were adjusted, and the logistic regression equation was reconstructed with sex, length of service, hazard factors, industry category, smoking status, and alcohol consumption as independent variables. Logistic regression analysis revealed that sex, length of service, hazard factors, industry category, smoking, and alcohol consumption are independent influencing factors of ONIHL. The risk of ONIHL in females is 0.496 (0.347–0.709) times greater than that in males. For each additional year of service in noise-exposed work, the risk of developing ONIHL increases by 1.045 (1.031–1.058) times. The risk of ONIHL in the noise-dust group and other groups is 1.271 (1.011–1.597) times and 0.619 (0.479–0.800) times greater than that in the noise exposure group, respectively. The risk of ONIHL in the manufacturing industry and other industries is 2.085 (1.336–3.254) and 2.063 (1.060–4.012) times greater than that in the energy production and supply industry, respectively. The risk of ONIHL in occasional smokers and regular smokers is 0.863 (0.652–1.142) and 1.216 (0.987–1.497) times greater than that in nonsmokers, respectively, but the differences are not statistically significant. The risk of ONIHL in excessive drinkers was 2.171 (1.476–3.193) times greater than that in nondrinkers (Table 4).

**Table 4:** Logistic regression analysis of factors affecting ONIHL.

Variables	*β*	S.E.	Wald	*p*	OR	95% CI
Sex			–	–		
Male	–	–	–	–	1.000	–
Female	−0.702	0.182	14.784	0.000	0.496	0.347–0.709
Years of exposure to hazards (years)	0.044	0.007	44.987	0.000	1.045	1.031–1.058
Hazard factors			33.171	0.000		
Noise	–	–	–	–	1.000	–
Noise and dust	0.240	0.117	4.229	0.040	1.271	1.011–1.597
Welding	−0.063	0.159	0.157	0.692	0.939	0.687–1.283
Noise and high temperature	0.081	0.302	0.073	0.787	1.085	0.600–1.961
Other groups	−0.479	0.131	13.403	0.000	0.619	0.479–0.800
Industry category			14.161	0.007		
Energy production and supply	–	–	–	–	1.000	–
Scientific research and technical services	0.531	0.286	3.443	0.064	1.700	0.971–2.979
Wholesale and retail	0.305	0.303	1.017	0.313	1.357	0.750–2.457
Manufacturing	0.735	0.227	10.470	0.001	2.085	1.336–3.254
Other industries	0.724	0.339	4.549	0.033	2.063	1.060–4.012
Smoking			6.806	0.033		
Non-smokers	–	–	–	–	1.000	–
Occasional smokers	−0.148	0.143	1.064	0.302	0.863	0.652–1.142
Regular smokers	0.195	0.106	3.377	0.066	1.216	0.987–1.497
Drinking			15.549	0.000		
Non-drinkers	–	–	–	–	1.000	–
Moderate drinkers	0.109	0.102	1.140	0.286	1.116	0.913–1.364
Excessive drinkers	0.775	0.197	15.512	0.000	2.171	1.476–3.193

### 3.3 Detection status of occupational hazard factors in three manufacturing enterprises

Among the three manufacturing enterprises, A is a large specialized equipment manufacturing enterprise, B is a large beverage manufacturing enterprise, and C is a medium-sized metal product manufacturing enterprise. The median and interquartile range of fixed-position noise detection results for Company A, as well as the mean and standard deviation for Companies B and C, are 79.20 (8.50) dBA, (81.70 ± 6.54) dBA, and (79.50 ± 5.61) dBA, respectively. The noise level exceedance rates (with respect to 85 dBA) for these position are 19.39, 26.92, and 20.90%, respectively. The differences in the noise level detection results and exceedance rates among the three companies are not statistically significant.

The on-site investigation of Company A revealed that the types of dust include welding fumes, grinding wheel dust, and other particulate dust. The results of total dust concentration measurements from 33 dust sampling points showed that the 8-h time-weighted average concentration (8hr-TWA) of airborne dust ranged from 0.330 to 5.200 mg/m^3^, with a median and interquartile range of 1.060 (0.900) mg/m^3^. All measurement results were below the occupational exposure limits for these types of dust.

### 3.4 Comparison of pure-tone audiometry results before and after enrollment among noise-exposed workers in three enterprises

#### 3.4.1 Speech frequency hearing threshold

The median and interquartile range of the 500 Hz hearing threshold in both ears of noise-exposed workers at initial enrollment, the 1st-year, the 2nd-year, and the 3rd-year were 33.00 (10.00) dB HL, 35.00 (10.00) dB HL, 33.00 (12.00) dB HL, and 29.00 (10.00) dB HL, respectively. The differences between the 3rd-year and initial enrollment, the 1st-year, and the 2nd-year were statistically significant (*p*<0.05). The median and interquartile range of the 1,000 Hz hearing threshold in both ears of noise-exposed workers at initial enrollment, the 1st-year, the 2nd-year, and the 3rd-year were 35.00 (10.00) dB HL, 35.00 (13.00) dB HL, 33.00 (8.00) dB HL, and 33.00 (7.00) dB HL, respectively. The differences between the 3rd-year and initial enrollment, the 1st-year, and the 2nd-year were statistically significant. In addition, the difference between the 1st-year and initial enrollment was statistically significant (*p*<0.05). The median and interquartile range of the 2000 Hz hearing threshold in both ears of noise-exposed workers at initial enrollment, the 1st-year, the 2nd-year, and the 3rd-year were 30.00 (10.00) dB HL, 30.00 (13.00) dB HL, 30.00 (7.00) dB HL, and 30.00 (7.00) dB HL, respectively. There were no significant differences between the results at different enrollment years (*p*>0.05) (Table 5).

**Table 5:** Pure tone audiometry results at various frequencies for different enrollment times.

Group	Binaural 500 Hz (dB HL)	Binaural 1,000 Hz (dB HL)	Binaural 2000 Hz (dB HL)	Binaural 3,000 Hz (dB HL)	Binaural 4,000 Hz (dB HL)	Binaural 6,000 Hz (dB HL)
Initial enrollment	33.00 (10.00) a	35.00 (10.00) a	30.00 (10.00)	30.00 (10.00) a	35.00 (10.00) a	38.00 (15.00) a
1st year	35.00 (10.00) a	35.00 (13.00) b	30.00 (13.00)	31.00 (15.00) b	36.00 (15.00) b	40.00 (14.00) b
2nd year	33.00 (12.00) a	33.00 (8.00) a.b	30.00 (7.00)	30.00 (10.00) a.b	35.00 (14.00) b	40.00 (16.00) b
3rd year	29.00 (10.00) b	33.00 (7.00) c	30.00 (7.00)	30.50 (10.00) b	36.00 (15.00) c	37.00 (14.00) a
*Z* value	95.404	40.011	7.059	14.492	41.693	61.497
*p* value	<0.001	<0.001	0.070	0.002	<0.001	<0.001

Identical letters indicate no significant difference between the two groups.

#### 3.4.2 High frequency hearing threshold

The median and interquartile range of the 3,000 Hz hearing threshold in both ears of noise-exposed workers at initial enrollment, the 1st-year, the 2nd-year, and the 3rd-year were 30.00 (10.00) dB HL, 31.00 (15.00) dB HL, 30.00 (10.00) dB HL, and 30.50 (10.00) dB HL, respectively. The differences between the initial enrollment and the 1st-year, the 3rd-year were statistically significant (*p*<0.05). The median and interquartile range of the 4,000 Hz hearing threshold in both ears of noise-exposed workers at initial enrollment, the 1st-year, the 2nd-year, and the 3rd-year were 35.00 (10.00) dB HL, 36.00 (15.00) dB HL, 35.00 (14.00) dB HL, and 36.00 (15.00) dB HL, respectively. The differences between the initial enrollment and the 1st-year, the 2nd-year, and the 3rd-year were statistically significant. In addition, the difference between the 3rd-year and the 1st-year, the 2nd-year were statistically significant (*p*<0.05). The median and interquartile range of the 6,000 Hz hearing threshold in both ears of noise-exposed workers at initial enrollment, the 1st-year, the 2nd-year, and the 3rd-year were 38.00 (15.00) dB HL, 40.00 (14.00) dB HL, 40.00 (16.00) dB HL, and 37.00 (14.00) dB HL, respectively. The differences between the initial enrollment and the 1st-year, the 2nd-year were statistically significant. In addition, the difference between the 3rd-year and the 1st-year, the 2nd-year were statistically significant (*p*<0.05) (Table 5).

### 3.5 The impact of combined noise and dust exposure on HFHL in workers

Pure-tone audiometry detected 2 cases (0.55%) of SFHL, with insufficient abnormal results to meet the requirements of the generalized estimation equation.

Pure-tone audiometry detected 163 cases (11.26%) of HFHL. Using HFHL detection (0 = no, 1 = yes) as the dependent variable and age at enrollment, length of service at enrollment, enrollment duration, and hazardous factors as independent variables, a generalized estimation equation was constructed. The results revealed that enrollment duration and hazardous factors influenced HFHL occurrence. Compared with that at the time of enrollment, the risk of developing HFHL after the 1st, 2nd, and 3rd years was 1.540 (1.128–2.103) times, 1.994 (1.409–2.821) times, and 1.628 (1.170–2.264) times greater, respectively. The risk of developing HFHL in the noise and dust exposure group was 4.660 (1.584–13.711) times greater than that in the noise exposure group (Table 6).

**Table 6:** Generalized estimating equation analysis of factors affecting HFHL.

Variables	𝛽	S.E.	Wald	*p*	OR	95%CI
(Intercept)	−4.167	0.527	62.447	<0.001	0.015	0.006–0.044
Noise and dust group	1.539	0.551	7.815	0.005	4.660	1.584–13.711
≥35 Years of age group	0.461	0.297	2.41	0.121	1.585	0.886–2.836
>8 Years of exposure duration	0.212	0.361	0.347	0.556	1.237	0.610–2.507
1st year	0.432	0.159	7.376	0.007	1.540	1.128–2.103
2nd year	0.69	0.177	15.202	<0.001	1.994	1.409–2.821
3rd year	0.487	0.168	8.377	0.004	1.628	1.170–2.264

The reference groups are the noise exposure group, <35 Years Group, ≤8 Years group, and initial enrollment.

### 3.6 The impact of enrollment time on hearing loss at different frequencies

Using the generalized estimating equation (GEE) to analyze the effect of the enrollment time on the detection rates of hearing loss at different high-frequency hearing thresholds (3,000 Hz, 4,000 Hz, 6,000 Hz). The risk of 3,000 Hz hearing loss in the 1st-year, the 2nd-year, and the 3rd-year were 1.578 (0.869–2.864) times, 1.461 (0.810–2.635) times, and 2.294 (1.395–3.771) times higher than at the initial enrollment, respectively. The difference between the 3rd-year and the initial enrollment was statistically significant (*p* < 0.05). The risk of 4,000 Hz hearing loss in the 1st-year, the 2nd-year, and the 3rd-year were 1.451 (1.097–1.920) times, 1.672 (1.212–2.307) times, and 2.141 (1.591–2.882) times higher than at the initial enrollment, with all differences being statistically significant (*p* < 0.05). The risk of 6,000 Hz hearing loss in the 1st-year, the 2nd-year, and the 3rd-year were 1.445 (1.087–1.919) times, 2.071 (1.581–2.713) times, and 1.415 (1.052–1.905) times higher than at the initial enrollment, with all differences being statistically significant (*p* < 0.05) (Table 7).

**Table 7:** The impact of enrollment time on hearing loss at different frequencies.

Frequencies	Time	𝛽	S.E.	Wald	*p*	OR	95%CI
3000Hz	Initial enrollment	-	-	-	-	1.000	-
	1st year	0.456	0.304	2.248	0.134	1.578	0.869-2.864
	2nd year	0.379	0.301	1.588	0.208	1.461	0.810-2.635
	3rd year	0.830	0.254	10.715	0.001	2.294	1.395-3.771
4000Hz	Initial enrollment	-	-	-	-	1.000	-
	1st year	0.372	0.143	6.810	0.009	1.451	1.097-1.920
	2nd year	0.514	0.164	9.809	0.002	1.672	1.212-2.307
	3rd year	0.761	0.152	25.200	<0.001	2.141	1.591-2.882
6000Hz	Initial enrollment	-	-	-	-	1.000	-
	1st year	0.368	0.145	6.442	0.011	1.445	1.087-1.919
	2nd year	0.728	0.138	27.921	<0.001	2.071	1.581-2.713
	3rd year	0.347	0.152	5.256	0.022	1.415	1.052-1.905

The reference groups are the initial enrollment.

## 4 Discussion

NIHL is the second most common type of sensorineural hearing loss after age-related hearing loss (27, 28). NIHL is primarily detected via pure tone audiometry, with test frequencies divided into speech frequencies (500, 1,000, 2000 Hz) and high frequencies (3,000, 4,000, 6,000 Hz). A cross-sectional study reported that the prevalence of SFHL is related to age, sex, and family history of deafness but not to noise exposure (29). Some studies have shown that the risk of SFHL is not significantly associated with sex, noise intensity, peak level, or exposure duration but is only associated with the age of the subject (30). Our study results show that the prevalence of SFHL among noise-exposed workers is related only to age, with an increased prevalence as age increases, which is consistent with the findings of the aforementioned studies.

HFHL is an important characteristic of NIHL (31, 32), which develops slowly and exhibits bilateral symmetry (33). Our study revealed that demographic characteristics such as sex and age are associated with the prevalence of HFHL, with males having a higher prevalence than females do, and the prevalence of HFHL increases with age, which is consistent with previous research findings (30). In terms of hazard factors, the prevalence of HFHL was greater in the noise dust group and the welding group than in the noise exposure group. After adjusting for potential confounding factors, hazards remained independent factors affecting HFHL, with welders having a prevalence 1.440 times higher than those exposed to noise alone. Welders experience combined exposure to welding fumes and noise, with research suggesting that welding fume exposure can increase the sensitivity of the autonomic nervous system to noise (34). There are significant differences in the prevalence of HFHL among employees in different industry categories and sizes of enterprises, with a higher prevalence in the wholesale, retail, and manufacturing industries. Small and microenterprises have higher prevalence rates than do large and medium-sized enterprises. After adjusting for potential confounding factors, industry category and enterprise size remained independent factors influencing HFHL.

GBZ49-2014, “Diagnosis of occupational noise-induced deafness,” sets the diagnostic criterion for ONIHL as a bilateral high-frequency average hearing threshold of ≥40 dB HL (35). Our study revealed that the prevalence of ONIHL is greater in males than in females, increases with age and years of service, and is significantly greater among workers who smoke and drink, which is consistent with some research conclusions (30, 36, 37). This may be related to the larger number of male workers, longer and more intense noise exposure, and increased risk of age-related hearing loss due to cochlear synaptic lesions, reactive oxygen species, and mitochondrial abnormalities. Unhealthy habits such as smoking and drinking in males further exacerbate hearing loss through combined effects with noise. In terms of hazard factors, the detection rates of ONIHL are higher in the noise-dust, welding, and noise-high-temperature groups than in the noise exposure group. After adjusting for potential confounders, these hazard factors remained independent influencing factors of ONIHL, with workers exposed to both noise and dust having an ONIHL prevalence 1.271 times higher than those exposed to noise alone. A cross-sectional study in Brazil investigated the hearing status of 4,875 workers and reported that 7.0% self-reported hearing impairment, with dust-exposed workers having a 1.77 times greater risk of hearing loss than those not exposed to dust (38). The prevalence of ONIHL is higher in manufacturing, and significantly greater in small and micro enterprises than in large and medium enterprises. After adjusting for potential confounders, industry category remained an independent influencing factor for ONIHL. The reasons may be related to the large number of workers in manufacturing, complex production processes leading to widespread noise sources, and higher levels of noise exposure. Small and microenterprises often have outdated production processes, minimal investment in occupational disease prevention, and a lack of occupational health management systems due to financial and technical constraints (31), resulting in multiple and severe occupational hazard factors.

Cross-sectional studies cannot establish causal relationships between exposure factors and outcomes, and the lack of baseline data often leads to bias. Therefore, we designed a longitudinal study to investigate the effects of noise exposure on the auditory system of workers and its combined effects with dust. The results revealed that the speech frequency hearing thresholds of noise-exposed workers fluctuated over different enrollment years but showed no clear trend. Pure tone audiometry is a subjective test, and its results can directly affect workers’ occupational health examination conclusions. Workers may be motivated to perform better in pure tone audiometry, and with repeated tests, some may have learned how to achieve better results. Therefore, the fluctuation in speech frequency hearing thresholds cannot yet be attributed to noise exposure. During the follow-up period, the prevalence of speech frequency hearing loss among noise-exposed workers was low. The reason is that the speech frequency hearing threshold results significantly impact the suitability assessment conclusions. A pure-tone air conduction hearing threshold >25 dB at 500 Hz, 1,000 Hz, or 2000 Hz is considered an occupational contraindication for noise-exposed positions (39). Therefore, the healthy worker effect appears.

HFHL is an early manifestation of noise exposure (40), with 4,000 Hz being the most susceptible to noise. The “4,000 Hz hearing notch” has been widely accepted as a characteristic feature of noise-induced hearing loss (41–43). The results of this study showed that the risk of 4,000 Hz hearing loss in noise-exposed workers increased with the duration of enrollment, confirming the above findings. However, studies on HFHL risk indicated that the odds ratio peaked in the second year. We speculate this counterintuitive result is mainly due to HFHL encompassing the frequencies of 3,000 Hz, 4,000 Hz, and 6,000 Hz, with the hearing threshold at 6000 Hz having a higher measurement error (44), which affected the final result.

Studies on the health effects of dust exposure on workers have focused mostly on the respiratory system, with some attention given to cardiovascular system damage (45–47). There are few studies on the effects of dust exposure on the auditory system. With the development of productivity and technological innovation, the exposure scenarios of hazardous factors in the workplace are becoming increasingly complex, and the impact of combined exposure to hazardous factors on workers’ health is receiving increasing attention. Cross-sectional studies of domestic populations seem to suggest that combined exposure to noise and dust can increase the risk of hearing loss among workers, which is consistent with the cross-sectional findings of this study. To further investigate the impact of combined exposure to noise and dust on hearing loss, we designed a longitudinal study. By analyzing the pure-tone audiometry results of subjects over four consecutive years, we concluded that enrollment years and hazard factors are independent influencing factors for high-frequency hearing loss among noise-exposed workers. The risk of high-frequency hearing loss increased after enrollment compared with the time of enrollment, and the overall trend increased with the number of years enrolled, suggesting a dose–response relationship between noise exposure and high-frequency hearing loss.

This study revealed that workers exposed to both noise and dust have a significantly greater risk of high-frequency hearing loss than workers exposed to noise alone do, suggesting a combined effect of noise and dust in causing high-frequency hearing loss. The mechanism of this combined effect is currently unclear, but it is speculated to be related to oxidative stress. Noise exposure has been confirmed to cause oxidative stress in cochlear tissue, and reactive oxygen species (ROS), which are important products, are considered closely related to noise-induced hearing loss (48). Dust exposure can also induce oxidative stress (49, 50), as reactive groups on the surface of dust entering the body can trigger respiratory bursts in macrophages, resulting in the formation of large amounts of ROS. Previous studies have suggested that promoting oxidative stress may increase the risk of noise-induced hearing loss (51). The validity of the above viewpoints requires further basic research for verification.

## 5 Conclusion

The number of workers exposed to noise in the manufacturing industry is high, and their hearing loss is more severe than that in other industries. The number of employees in microenterprises undergoing occupational health examinations is significantly lower than that in enterprises of other scales, and the prevalence of ONIHL increases with decreasing enterprise size. Therefore, the manufacturing industry and microenterprises should be the main targets of occupational health supervision. Workers exposed to noise predominantly suffer from HFHL. Combined exposure to noise and dust is a risk factor for ONIHL. In addition, ONIHL is influenced by gender, years of service, industry type, smoking, and alcohol consumption. Employers should strengthen occupational health management, improve workers’ occupational health literacy, focus on older male workers with long service periods and those with bad habits such as smoking and drinking, and advise them to quit. Combined exposure to noise and dust may have a synergistic effect on causing HFHL. Comprehensive protective measures should be taken for workers with combined exposure.

## Data Availability

Requests to access the datasets should be directed to jiaxiang5337@ahmu.edu.cn.
